# Cognitive reserve and neural oscillations in face perception and memory processing in patients with MCI and AD

**DOI:** 10.1002/alz70857_102451

**Published:** 2025-12-25

**Authors:** Yingxin Mi, Changming Wang, Lei Liu, Aonan Li, Yi Tang

**Affiliations:** ^1^ Department of Neurology & Innovation Center for Neurological Disorders, Xuanwu Hospital, Capital Medical University, National Center for Neurological Disorders, Beijing, Beijing, China; ^2^ Department of Neurosurgery Xuanwu Hospital, Capital Medical University, Beijing, Beijing, China; ^3^ Neurodegenerative Laboratory of Ministry of Education of the People's Republic of China, Beijing, Beijing, China

## Abstract

**Background:**

Prosopagnosia is a significant symptom observed in the middle and late stages of Alzheimer's disease (AD), which severely affects patients and their families' daily life. Our study employed an occluded face delay matching paradigm (OFDM) to investigate the progression of face perception and memory, and the underlying neural mechanisms in individuals with healthy aging, mild cognitive impairment (MCI), and AD.

**Method:**

We enrolled 180 participants, including 60 cognitively normal (CN) individuals, 69 MCI, and 51 AD patients. We combined the OFDM task and electroencephalogram (EEG) recordings to explore the temporal dynamics of face encoding, maintenance, and retrieval. Participants also underwent neuropsychological assessments and testing for cerebrospinal fluid amyloid and tau markers.

**Result:**

Behavioral performance shows a clear gradient descent across the three groups, diagnostic models based on task performance yielded an area under the curve (AUC) value of 0.79 (95% CI: 0.72‐0.86, *p* < 0.001). The EEG results further revealed that in patients with AD, impairments in face processing were associated not only with compromised face memory (the negative slow wave, NSW: *p* = 0.0029) but also with deficits in both early (P1: *p* = 0.0277) and later stages of face processing (N170: *p* = 0.0335, P2: *p* = 0.0149). More importantly, face impairment in AD continuum was already detectable in the MCI stage. Patients with MCI showed abnormalities in early face encoding (P1: *p* = 0.0277) and perception (N170: *p* = 0.0335), yet they preserved certain higher‐level face processing abilities (P2: *p* = 0.3016) and aspects of face memory (NSW: *p* = 0.0974). Furthermore, impaired face‐perception in MCI and AD was characterized by decreased theta and alpha band power and synchronization. The preservation of face memory in MCI was linked to the activation of alpha power. Additionally, the adverse impact of T‐Tau on Mini‐Mental State Examination (MMSE) scores was partially mediated by theta oscillation (indirect effect = 0.00185, 95% CI: 0.000148 ‐ 0.028).

**Conclusion:**

This study indicates that our OFDM task effectively differentiates between different stages of cognitive impairment. As the pre‐stage of AD, MCI patients have relatively preserved high‐level face cognition and face memory despite early face processing deficits, highlighting potential targets for early intervention and neural regulation in AD.

